# Outdoor Air Emissions, Land Use, and Land Cover around Schools on Tribal Lands

**DOI:** 10.3390/ijerph16010036

**Published:** 2018-12-24

**Authors:** Nirmalla Barros, Nicolle S. Tulve, Ken Bailey, Daniel T. Heggem

**Affiliations:** 1 Oak Ridge Institute for Science and Education, U.S. Environmental Protection Agency, Office of Research and Development, National Exposure Research Laboratory, 109 T.W. Alexander Drive, Mail Code: E205-04, Research Triangle Park, NC 27709, USA; 2 U.S. Environmental Protection Agency, Office of Research and Development, National Exposure Research Laboratory, 109 T.W. Alexander Drive, Mail Code: E205-04, Research Triangle Park, NC 27709, USA; tulve.nicolle@epa.gov; 3 U.S. Environmental Protection Agency, Office of Research and Development, Office of Science Policy, 3355 Blue Rock Road, Cincinnati, OH 45239, USA; envsci@zoomtown.com; 4 U.S. Environmental Protection Agency, Office of Research and Development, National Exposure Research Laboratory, 944 East Harmon Avenue, Las Vegas, NV 89119, USA; motoheggem2@gmail.com

**Keywords:** children, school, American Indian/Alaska Native, air pollution, land use, land cover

## Abstract

Children from tribes are more burdened with adverse respiratory well-being outcomes versus other U.S. children. The objectives of this study were to identify stressors from the built and natural environments for tribal school-aged children. Outdoor air concentrations around U.S. tribal schools were linked to National Emission Inventories; ecoregions and National Land Cover Database; and American Community Survey and school map layers. Nine school sites (seven tribes, five U.S. states) were in three ecoregions: North American Deserts, Northern Forests, and Mediterranean California. Closest emission sources were oil, gas, airport, and manufacturing facilities. Maximum annual outdoor air concentrations were measured for toluene at two schools (29 ppb and 15 ppb, 2011), located four miles from a solid waste landfill and eight miles from paperboard/saw mills. Maximum annual concentrations of metals in particulate matter 10 micrometers and smaller were highest for manganese (68 ng/m^3^, 2011). Schools were in mainly arid and heavily forested lands. Closest emission sources were predominantly off tribal lands. Measurements were limited (<30/year). Compared to schools off tribal lands, schools on tribal lands were further away from roadway sources. Future research may examine outdoor air quality around schools with more developed land and indoor air for tribal children’s total exposure.

## 1. Introduction

Compared to adults, children are more vulnerable to exposure from both chemical and non-chemical stressors found in their everyday environment, leading to differences in their health and well-being. In the U.S., children from tribal communities have a greater burden of adverse health and well-being outcomes versus children from other communities [1]. According to the U.S. Department of Health and Human Services, in 2015 American Indian/Alaska Native (AI/AN) children had a 60% higher likelihood of having asthma compared to non-Hispanic White children [2]. For AI/AN children between the ages of 1 and 14 years old, the leading cause of hospitalizations was respiratory disease [1].

Distinct stressors from AI/AN children’s everyday environment may contribute to this increased burden of adverse respiratory health and well-being outcomes and their exposures to environmental contaminants. A stressor is characterized as any physical, chemical, social, or biological entity [3]. A state-of-the-science review [4] targeted stressors impacting AI/AN children’s health and well-being, specifically from their built (i.e., man-made) and natural environments.

Twelve studies were identified that evaluated AI/AN children’s risk of respiratory illness from stressors in their built environment [5,6,7,8,9,10,11,12,13,14,15,16]. Findings from this review suggested there is an increase of respiratory illness among AI/AN children resulting from two non-chemical stressors, indoor use of wood for cooking or heating or lack of indoor plumbing. For indoor use of wood for cooking or heating, we observed an increase in lower respiratory tract infections (LRTIs) [6,9]; cough between colds [11]; acute lower respiratory infections (ALRIs) [13]; and pneumonia [14]. For lack of indoor plumbing, we observed an increase in LRTIs [5,6,7,8,9]; pneumococcal colonization [10]; and invasive pneumococcal disease [15]. From our review, the only chemical stressors identified as being associated with a higher risk of respiratory illness were from volatile organic compounds (VOCs) (benzene, toluene, ethylbenzene, o-xylene and m,p-xylene (BTEX) >100 µg/m^3^), PM_2.5_ (> 25 µg/m^3^) [11], or respirable particles (≥65 µg/m^3^) [13] in homes using wood for cooking or heating. An increased risk was found for cough between colds (odds ratio (OR): 4.42, *p* < 0.001), wheezing between colds (OR: 1.88, *p* = 0.068), an asthma diagnosis (OR: 3.02, *p* = 0.031) [11], or ALRIs (OR: 7.0, 95% confidence interval (CI): 0.9–56.9) [13]. Other identified studies found increased risks of respiratory illness from mold [LRTIs: 6, flu: 14] or outdoor air quality concerns from outdoor smoke [14] or a burn-barrel near the home [12], but these findings were not statistically significant.

Our review [4] also identified stressors from AI/AN children’s natural environment around where they lived. Among the 21 studies that identified stressors from their natural environment, 19 studies reported on the same non-chemical stressor: residential proximity to polluted landscapes. Twelve of these 19 studies [17,18,19,20,21,22,23,24,25,26,27,28] evaluated the impact of residential proximity to polluted landscapes on developmental outcomes, one of which examined lung function among adolescents [17]. Significant associations were found between increased closing volume (potentially suggestive of lung abnormalities in small airways) among male adolescents who had lived near an aluminum smelter compared to those who had lived farther away from the smelter and increased levels of urinary fluoride [17]. Other studies found associations between residential proximity to hazardous waste sites among AI/AN adolescents. Risk of exposure was from polychlorinated biphenyls (decreased cognitive function [19,20]; reduction in thyroid function [21]; or elevated risk of autoimmune disease [22]), as well as relationships between lead and delays in sexual maturation among female adolescents [18].

However, an information gap was identified surrounding chemical and non-chemical stressors impacting AI/AN children’s health and well-being beyond their households [4]. These 35 studies only targeted stressors from where AI/AN children lived [4]. A child’s total environment, which also includes where children learn and play, needs to be considered when examining the interrelationships between children’s inherent characteristics (e.g., sex, genetics) and their activities and behaviors in influencing their exposures to chemical and non-chemical stressors and the impact of these stressors on children’s health and well-being [3]. For this study, we aimed to reduce the information gap we identified in our state-of-the-science review [4] by examining chemical and non-chemical stressors for AI/AN children around their schools’ built and natural environments.

Our objectives were to: (1) identify the extent and type of land use and land cover (LULC) surrounding these schools; (2) identify the major sources of outdoor air emissions near these schools; (3) describe schools’ outdoor air quality conditions; and (4) identify school-age populations and number of schools impacted by these conditions on U.S. tribal lands.

## 2. Materials and Methods

### 2.1. School Selection from Available Outdoor Air Monitoring

Tribal schools in Colorado and Idaho were selected from the more than 60 schools in 22 states that participated in the U.S. EPA’s School Air Toxics Monitoring Initiative (SATMI). For this initiative, schools were prioritized nationwide for an initial screening of potential impacts from toxic air pollution based on a newspaper (USA Today) analysis, which applied U.S. EPA’s Risk-Screening Environmental Indicators Model and the 2005 Toxics Release Inventory, and U.S. EPA’s 2002 National Air Toxics Assessment, then refined with additional and updated information, mainly provided by local and state air quality agencies [29]. The purpose of this initiative was to provide the basis for additional actions (e.g., continued monitoring, risk mitigation efforts) by the U.S. EPA, state, and local agencies.

After outdoor air pollutant monitoring was completed at these two tribal schools (Colorado and Idaho), the equipment used to perform the sampling was then distributed to other interested tribes for their schools [30]. As a result, we have available monitoring information for seven additional school sites on tribal lands [30]. For this study, we included nine school sites (two sites with monitoring done in 2009 and seven sites with monitoring done after 2009) using monitoring information that was already collected for SATMI.

### 2.2. Linkage of Nearby Outdoor Air Emission Sources, Outdoor Air Monitoring around Schools, Land Use and Land Cover, and Population and School Characteristics

#### 2.2.1. Nearby Outdoor Air Emission Sources

Sources of outdoor air emissions closest to schools were identified by linking school air monitor sites to the U.S. EPA’s National Emission Inventories (NEIs). NEIs provide estimates of air emissions from sources that emit criteria air pollutants and their precursors, as well as hazardous air pollutants based on air emission information reported from state, local, and tribal air agencies and data sources supplied by the U.S. EPA [31]. Criteria air pollutants include six common air pollutants (carbon monoxide, ground-level ozone, lead, nitrogen dioxide, particulate matter, and sulfur dioxide) for which the Clean Air Act requires that the U.S. EPA set National Ambient Air Quality Standards (NAAQS) [32]. Hazardous air pollutants are those that are known or suspected to cause cancer or other adverse health effects or adverse environmental effects [33].

#### 2.2.2. Outdoor Air Monitoring around Schools

The U.S. EPA’s SATMI targeted site-specific pollutants for initial monitoring lasting 60 to 90 days [29] (a quality assurance project plan was developed) [34]. Monitored pollutants varied at each school depending on available information about the pollution sources in the school area. Siting for the air sampling equipment was on school grounds. Air samplers were placed away from locations that were directly influenced by nearby school-based biasing emission sources (e.g., school-bus idling sites, backup generators, boiler stacks) [29]. Meteorological monitoring equipment was provided by the U.S. EPA and measured wind speed and direction [35]. Schools in Colorado and Idaho, for instance, were found to have similar wind patterns over the entire sampling period and similar to historical patterns at the same wind measurement station [35]. Outdoor air monitoring information for schools on tribal lands was obtained through the U.S. EPA’s Air Quality System [36].

#### 2.2.3. Land Use and Land Cover

To understand regional patterns of spatially-variable conditions that may influence outdoor air emissions around these schools, combinations of factors that include characteristics of soils, vegetation, physical geography, and climate were used to define ecoregions [37]. Ecoregions around schools were described using broad (Level I) ecoregions for North America [38]. Site-specific conditions for characterizing the land surface(s) around schools were obtained from the National Land Cover Database by subwatershed (hydrologic unit code 12) where each school’s outdoor air monitor was located and measures of land use diversity via U.S. EPA’s EnviroAtlas tool.

#### 2.2.4. Population and School Characteristics

To identify school-age populations impacted by these outdoor air conditions, population density and population (total, AI/AN, and school-age) estimates were obtained for the census tract around the selected locations of school air monitors from the 2008–2012 American Community Survey dataset (via U.S. EPA’s EnviroAtlas). We defined school-age populations to be 5– < 18 years. Counts of school-age populations were computed to obtain the total population less than 18 years of age then subtracting the total population less than five years old within the same census tract. Also, to identify the number of schools affected by these conditions, schools around the air monitors were gathered by measuring the distance from the location of the air monitor to the nearby school (includes day care centers, private schools, and public schools within a mile), which were identified via U.S. EPA’s Community-Focused Exposure and Risk Screening Tool (school layers were authored by U.S. EPA’s Geospatial Support (NGS) Team) [39].

## 3. Results

### 3.1. School Selection from Available Outdoor Air Monitoring

**Figure 1:**
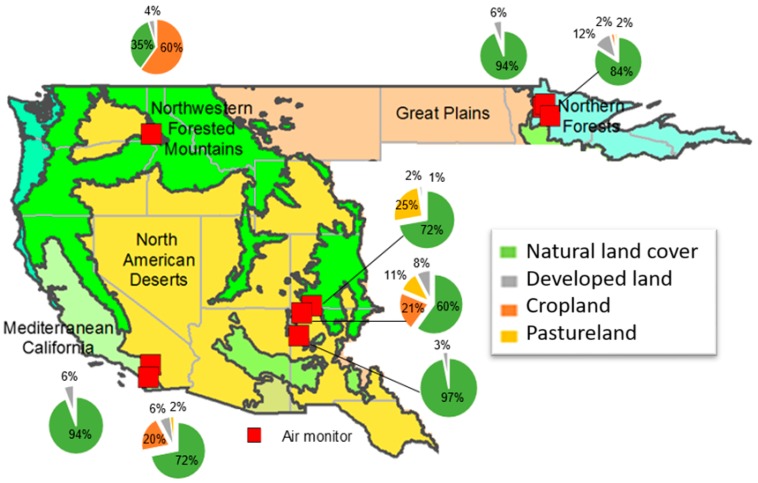
Land use and land cover around school air monitors.

For this study, outdoor air monitoring information was available for nine sites from seven tribes (Leech Lake Band of Ojibwe, Morongo Band of Mission Indians, Navajo Nation, Nez Perce Tribe, Red Lake Band of Chippewa Indians, Rincon Band of Luiseño Indians, and Southern Ute Indian Tribe) in five U.S. states (California (CA), Colorado (CO), Idaho (ID), Minnesota (MN), and New Mexico (NM)) (Figure 1, Table 1). School air monitors were situated in multiple counties in three states (CA, MN, and NM). The Navajo Nation (NM) and Red Lake of Chippewa Indians (MN) had air monitors in more than one location (two distinct sites each). Earliest monitoring started in 2006 at a Morongo Band of Mission Indians (Morongo) school in California (Riverside county) (Supplemental Material: Table S1). Ambient conditions at this school site were monitored the most (over 11 years and for five pollutants). It was the only ambient monitoring site to sample for criteria pollutants. The remaining eight school locations were monitored for metals in PM_10_ and VOCs. Among these eight school locations, sites on Red Lake Band of Chippewa Indians (Red Lake) (MN Beltrami county) and Navajo Nation (NM McKinley county) reservations were the most frequently monitored.

### 3.2. Linkage of Nearby Outdoor Air Emission Sources to Schools, Outdoor Air Monitoring around Schools, LULC, and Population and School Characteristics

#### 3.2.1. Nearby Outdoor Air Emission Sources and Outdoor Air Monitoring

Almost all closest sources of outdoor air emissions linked to these monitoring sites were located off tribal lands (Table 2). Using the NEI database, the closest source of air emissions was from a crude petroleum and natural gas extraction facility located within a mile of the school monitoring site on the Southern Ute Indian Tribe’s land (CO). NO_2_ (nitrogen dioxide), ozone, and PM_2.5_ were measured at only one school site on the Morongo Indian reservation (CA Riverside county) (Table S1). Within three miles of this school’s location were two outdoor air emission sources (i.e., an airport and a concrete manufacturing plant). Between 2006 and 2016, for Morongo’s monitoring location, daily maximum hourly values of annual mean ozone concentrations were between 0.06 and 0.07 ppm (maximum: 0.12–0.15) (NAAQS: 0.07 ppm, annual fourth-highest daily maximum 8-hour average concentration, averaged over three years). Over this 11-year monitoring period, there was more variation in annual mean concentrations of PM_2.5_ (between 8 and 14 µg/m^3^ (maximum: 57–395) (NAAQS: 12 µg/m^3^, annual mean, averaged over three years). Between 2015 and 2016, NO_2_ annual mean concentrations were between 2.7 and 3.3 ppb (maximum: 30–42) (NAAQS: 53 ppb, annual mean).

Maximum annual outdoor air concentrations for selected VOCs (benzene, toluene, ethylbenzene, and o-xylene) were measured by a monitor located at the Leech Lake school (MN Cass county) (toluene: 29 ppb in 2011, less than the sample screening level of 1062 ppb), a monitor located at the Nez Perce school (ID Nez Perce county) (toluene: 15 ppb in 2011), and a monitor at a Red Lake school (MN Beltrami county) (toluene: 12 ppb in 2011) (Table 3). Within four miles of these locations was a solid waste landfill (on tribal land), within eight miles was a paperboard and saw mill (off tribal land), and within 32 miles an airport (off tribal land), respectively. Measured annual median concentrations were greatest at a Navajo Nation (NM San Juan county) school monitoring site (toluene: 3.6 ppb in 2010), which was within three miles of a natural gas liquid extraction facility (off tribal land).

Annual maximum concentrations of metals in PM_10_ were highest for manganese measured at the Nez Perce school monitoring site (ID) (68 ng/m^3^ in 2011) (less than its sample screening level of 500 ng/m^3^; a sample result below this level is not expected to cause adverse health effects from short-term exposures) [35] (Table 4). This monitoring site also had the greatest maximum concentration of cobalt (1.5 ng/m^3^ in 2011) (less than the sample screening level of 100 ng/m^3^). For other metals, school monitoring sites in Minnesota had the highest maximum concentrations for chromium (Leech Lake (Cass county): 26 ng/m^3^ in 2011), nickel (Leech Lake (Cass county): 6.2 ng/m^3^ in 2011), and selenium (Red Lake (Beltrami county): 0.9 ng/m^3^ in 2011) (less than the sample screening levels of 200 ng/m^3^ for nickel and 20,000 ng/m^3^ for selenium). Annual beryllium, lead, and mercury median concentrations were low (less than 0.21 ng/m^3^ for lead and zero for beryllium and mercury) at six school monitoring sites (Morongo (CA Riverside), Nez Perce (ID), Red Lake (MN Beltrami Sites 1 and 2), Leech Lake (MN Cass), and Navajo Nation (NM McKinley)) (Table S2).

#### 3.2.2. Land Use and Land Cover

Air monitors located at school sites on tribal lands were situated in three distinct ecoregions: North American Deserts Ecoregion (represented by: arid areas lacking trees, comprised of unique shrub and cactus vegetation in an arid to semi-arid climate with seasonal temperature extremes; four monitors), Northern Forests Ecoregion (represented by extensive forests and lakes with numerous large drainage basin systems and a climate of long, cold winters and short, warm summers; three monitors), and Mediterranean California Ecoregion (represented by a mixture of lands including mountains, hills, and plains distinguished by a warm and mild Mediterranean climate; two monitors) [38] (Figure 1). Generally, these monitoring locations were in areas of low development (i.e., percent of developed land ranged from 2 to 12% by subwatershed). Except for one site for the Nez Perce reservation (ID) where percent of agricultural land was high (60% for cropland), the sites were in locations with heavy natural land cover (ranging from 60 to 97%). Natural land cover includes forest, grassland, barren land, shrubland, and wetland; across all sites, barren land was less than five percent of the natural land cover. Two school sites on the Navajo Nation reservation (NM) had very low diversity of land use types (0 to 20%) (a measure using job and housing unit counts by census block group), suggesting there was relatively little mix of land uses around these schools.

#### 3.2.3. Population and School Characteristics

**Figure 2:**
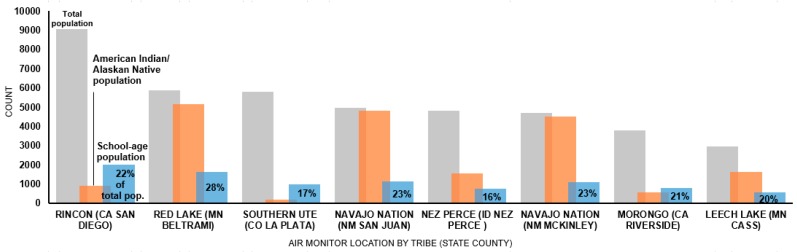
Population characteristics (by census tract) around monitor locations on tribal lands by greatest total population.

These schools were in census tracts with proportions of school-age populations (of the total population) ranging from 16% (Nez Perce ID) to 28% (Red Lake MN) (Figure 2). Sources of outdoor air emissions near these schools potentially impacted as many as seven schools located within a mile of air monitors in northwestern Minnesota (data not shown) and as many as 1648 school-aged children (28% of total population in census tract) being affected.

## 4. Discussion

To the best of our knowledge, this is the first evaluation to consider the links between outdoor air emissions, land use, and land cover and the combined impact on children attending school on U.S. tribal lands. Schools were included in this analysis based on their participation in the U.S. EPA’s SATMI. This initiative provided tribal decision-makers with an initial screening to promote healthy environments for AI/AN school-aged children (as well as faculty and staff in the schools). The monitoring was conducted by tribal staff with technical support from the Tribal Air Monitoring Support Center. There was extensive monitoring performed at a Morongo Band of Mission Indians school site (California), which was linked to two air emission sources within three miles from an airport and a concrete manufacturing plant. Besides this school, however, there were limited observations (<30) over a given year for pollutants at other schools.

Monitoring sites at schools on tribal lands were in areas of low development around arid and heavily forested lands. The major sources of outdoor air emissions closest to these schools were predominantly located off tribal lands. The closest source of air emissions was within a mile of a Southern Ute school (CO) (i.e., crude petroleum and natural gas extraction facility). Other potential sources of air emissions such as abandoned mines were not found to be in close proximity to these school sites, with the exception of one uranium mine on the Navajo Nation’s land (NM McKinley county) that was listed on the U.S. EPA’s Superfund Enterprise Management System [40].

Compared to sources of outdoor air emissions near schools on U.S. tribal lands, schools on non-tribal lands in U.S. cities were surrounded by more roadway sources like major highways [35,41] (Table 5) (non-tribal schools were selected based on their geographic proximity to the tribal schools). Schools on tribal and non-tribal lands within comparable U.S. regions in California and Minnesota were also surrounded by a mix of industrial sources like airports and manufacturing facilities (e.g., coatings and concrete manufacturing) [35].

Outdoor air sampling around schools on tribal lands had at times slightly greater average concentrations of metals in PM_10_ compared to schools on non-tribal lands [35] (Table 5). A school on Leech Lake land in Minnesota (Cass county) located within four miles of a solid waste landfill averaged 8.8 ng/m^3^ (3.5–26) for manganese and 1.6 ng/m^3^ (0.3–6.2) for nickel in 2011, but dropped to 2.5 ng/m^3^ for manganese in 2012 and rose to 2.7 ng/m^3^ for nickel in 2012. A nearby school on non-tribal land in the city of Minneapolis had slightly lower manganese (8.68 ng/m^3^, 1.19–21.5) and nickel (0.79 ng/m^3^, 0.15–4.06) concentrations when measured in 2009 [35]. The school was near a large municipal waste incinerator and a coatings manufacturing facility.

Air pollutants such as benzene, however, were similar for school sites located on tribal and non-tribal lands. For benzene, outdoor air concentrations were similar around a school on Rincon Band of Luiseño Indians land in California (San Diego county) (3.20 µg/m^3^ in 2012, 1.60–8.94) and a school on non-tribal land in El Paso, Texas (maximum median: 3.21 µg/m^3^ in 1999, 1.66–4.99) [41].

Other sampling studies demonstrated a greater concentration of outdoor air pollutants such as NO_2_ surrounding schools on non-tribal lands. Among 20 schools that were sampled in El Paso, TX around the international border between USA and Mexico [42], average NO_2_ concentrations were more than six times greater (20.6 ppb, 11.0–37.5) than concentrations around a school on tribal land (Morongo CA Riverside county) (2015 average 2.7 ppb (0–42), 2016 average 3.3 ppb (0–30)).

### 4.1. Limitations

The main limitation of this study was the limited number of sampling observations (<30) over a given year and few years of air monitoring of different pollutants at these schools. Some schools had more years of monitoring and more pollutants monitored based on site-specific factors (e.g., nearby air emission sources, availability of equipment). These different sampling patterns (e.g., by period) introduced more variation between the sampled sites. Also, this evaluation focused on major sources that were captured from the EPA’s NEI for hazardous air pollutants. Minor and area sources on tribal lands also contributing to air quality conditions for AI/AN school-age children were not available, which could have offered additional background about the nature of air pollution sources. Another limitation was the application of a convenience sample of schools that was limited to selection of schools that had available air monitoring information at the time this study was initiated. Schools preparing an application to propose monitoring on their lands, had already applied, were in the process of monitoring when our study started, or had completed monitoring after this study started, would not have been included in this study.

Despite these limitations, this analysis offers a background about the levels of pollution in outdoor air for schools that were located on tribal lands. These measured levels of air pollutants present information about pollutants commonly found in many areas in the USA. The amount of natural land cover near these schools was high and developed land was low. Levels of outdoor air pollution and the amount and types of industries nearby varied from school to school. These nearby sources of air emissions were predominantly off tribal lands, indicating that air pollution from sources off tribal lands also have implications for AI/AN school-aged children’s health and well-being. Tribal governments, however, do not have control over how these non-tribal lands are developed unless entities with jurisdiction over these lands consult them. AI/AN school-aged children’s potential exposures to stressors from their built environment were identified, providing insights for exposure and risk assessments and decision makers in understanding the interrelationships of these factors in influencing AI/AN children’s health and well-being.

According to the U.S EPA’s Report on the Environment, between 2012 and 2014, the prevalence of respiratory disease (asthma) among AI/AN children was 102 cases per 1000 children (following the rate among Black children—142 cases per 1000 children) (summarized from the Centers for Disease Control and Prevention’s National Health Interview Survey) [43]. By Indian Health Service area, in 2012, the Navajo service area had the highest admission rate for asthma hospitalizations (under age 18 years of age) (16.2 per 10,000 children) [44].

This greater burden of asthma occurrence among AI/AN children may be due less to outdoor emission sources and outdoor pollutant concentrations around their school environment that we identified in this analysis, but more to their built household environments. Their distinct household environment non-chemical and chemical stressors from the indoor use of wood for cooking or heating, which we described previously in Barros et al. [4], may play a larger role in impacting their risk of respiratory illness.

### 4.2. Future Research

Opportunities for future research to gather additional insights on potential stressors from around AI/AN children’s built environment beyond their households include examining LULC changes from one period to another (e.g., growing urban areas or decline) to assess development with increasing or decreasing emissions (for instance, related to oil and gas extraction activities). Other opportunities include expanding this initial monitoring to include schools on tribal lands in more developed areas (i.e., higher percentages of urban and agriculture lands to get insights about other air pollution sources from pesticides for crops, for instance) and expanding monitoring to indoor school environments to also evaluate children’s indoor school exposures (e.g., metals). To further understand potential stressors influencing AI/AN children’s exposures, other factors need to be considered that provide an evaluation of their environments’ resilience to control for outdoor air pollutants. These other factors can consider ecological conditions that provide an indication of sustainability and ability to control for stressors such as related to changes in soil/land patterns, vegetation (e.g., integrity of wetlands), and water use (e.g., pathogens). Additional studies can be done to link stressors from their built and natural environments with stressors from their social environments (e.g., tobacco smoking, family income, parental educational level) to evaluate their total environment and impacts on their health and well-being.

## 5. Conclusions

To the best of our knowledge, this is the first evaluation to link land use and land cover with outdoor air emissions near schools on U.S. tribal lands. Schools were predominantly in areas of low development around arid and heavily forested lands. Besides extensive monitoring around one school, there were limited observations of different pollutants over a given year at other schools. The closest sources of outdoor air emissions were mainly off tribal lands. Tribal governments do not typically control how lands near them are developed unless entities with jurisdiction over them consult them. The findings from this study can be used to direct future research efforts to further examine outdoor air quality around schools on tribal lands, especially, to expand monitoring to areas with more developed and/or agricultural land, as well as to monitor indoor air quality conditions in schools to provide total exposure information for AI/AN children.

## Figures and Tables

**Table 1:** Chronology of outdoor air measurements around schools by year, tribal monitoring location, and pollutant measured.

Year	2009		2010		2011		2012		2013		2014		2015	
Tribe (U.S. State County)	Metals in PM_10_	VOCs	Metals in PM_10_	VOCs	Metals in PM_10_	VOCs	Metals in PM_10_	VOCs	Metals in PM_10_	VOCs	Metals in PM_10_	VOCs	Metals in PM_10_	VOCs
Morongo Band of Mission Indians (CA Riverside) ^1^														
Rincon Band of Luiseño Indians (CA San Diego)														
Southern Ute (CO La Plata)														
Nez Perce (ID Nez Perce)														
Red Lake Band of Chippewa Indians (MN Beltrami Site 1)														
Red Lake of Chippewa Indians (MN Beltrami Site 2)														
Leech Lake Band of Ojibwe (MN Cass)														
Navajo Nation (NM McKinley)														
Navajo Nation (NM San Juan)														

PM_10_—particulate matter 10 micrometers and smaller; VOCs—volatile organic compounds; ^1^ also had sampling for nitrogen dioxide (2015), ozone (2006–2016), and particulate matter 2.5 micrometers and smaller (2006–2016). The blue color represents the years of measurement by pollutant for the respective school location.

**Table 2:** Major sources of air emissions closest to school air monitors.

Tribe (U.S. State County)	Distance of Closest Source of Emissions—Mile(s)	NAICS ^1^ Description	Major Sources of Emissions	Amount of Emissions/Year—ton(s)
Southern Ute (CO La Plata) ^2^	1 | on tribal land	Crude petroleum and natural gas extraction	NO_X_	89
			VOCs	12
			SO_2_	3
Navajo Nation (NM San Juan) ^2^	3 | off tribal land	Natural gas liquid extraction	SO_2_	621
			NO_X_	105
			CO	87
			VOCs	59
Navajo Nation (NM McKinley) ^3^	3 | off tribal land	Petroleum bulk stations and terminals	NOx	404
			CO	84
			VOCs	40
Morongo (CA Riverside) ^3^	3 | off tribal land	Other airport operations	CO	13
			VOCs	0.34
			PM_10_	0.27
	3 | off tribal land	Ready-mix concrete manufacturing	PM_10_	3
			PM_2.5_	0.59
			VOCs	0.001
Rincon (CA San Diego) ^3^	3 | off tribal land	Airport operations	CO	2
			PM_10_	0.04
			PM_2.5_	0.03
Leech Lake (MN Cass) ^2^	4 | on tribal land	Solid waste landfill	VOCs	0.47
			SO_2_	0.07
Nez Perce (ID Nez Perce) ^2^	8 | off tribal land	Paperboard mills	CO	4145
			NO_X_	1274
			VOCs	401
	8 | off tribal land	Sawmills	VOCs	50
			PM_10_	43
			PM_2.5_	15
Red Lake (MN Beltrami) ^2^	32 | off tribal land	Airport	CO	0.5
			PM_10_	0.01
			VOCs	0.007

^1^ North American Industry Classification System; ^2^ U.S. EPA’s 2011 National Emission Inventory (NEI); ^3^ U.S. EPA’s 2014 NEI; NO_X_—nitrogen oxides; VOCs—volatile organic compound; SO_2_—sulfur dioxide; CO—carbon monoxide; PM_10_—particulate matter 10 micrometers and smaller; PM_2.5_—particulate matter 2.5 micrometers and smaller.

**Table 3:** Summary of outdoor air concentrations (ppb) of volatile organic compounds by location and measurement year.

Monitor location—Tribe (U.S. State County)	Year	Compound (Sample Screening Level ^1^)	No. of Samples	Median (Mean ± Standard dev.)	Minimum–Maximum
Morongo (CA Riverside)	2014	B * (9 ppb)	11	0.5 (0.5 ± 0.2)	0.3–0.9
		T * (1061 ppb)	11	0.7 (0.9 ± 0.7)	0.3–2.7
		E * (9212 ppb)	11	0.2 (0.2 ± 0.09)	0.05–0.3
		X * (2073 ppb)	11	0.4 (0.4 ± 0.2)	0.1–0.6
Rincon (CA San Diego)	2012	B	9	0.8 (1.0 ± 0.7)	0.5–2.8
		T	9	2.9 (2.5 ± 1.1)	0.7–3.8
		E	9	0.5 (0.6 ± 0.2)	0.2–0.9
		X	9	1.2 (1.4 ± 0.8)	0.4–2.7
Southern Ute (CO La Plata)	2009	B	14	0.8 (0.9 ± 0.4)	0.4–1.5
		T	14	1.0 (1.0 ± 0.3)	0.4–1.6
		E	14	0.2 (0.1 ± 0.1)	0.04–0.2
		X	14	0.3 (0.3 ± 0.1)	0.2–0.6
Nez Perce (ID Nez Perce)	2011	B	10	0.7 (0.7 ± 0.2)	0.4–1.0
		T	10	1.4 (2.6 ± 4.3)	0.6–15
		E	10	0.3 (0.3 ± 0.2)	0.2–0.9
		X	10	0.5 (0.7 ± 0.5)	0.3–2.1
Red Lake (MN Beltrami)^2^	Site 1 (2010 | 2011), Site 2 (2011)	B	3 | 7, 10	1.2 (1.2 ± 0.3) | 1.2 (1.1 ± 0.2), 0.6 (0.7 ± 0.3)	1.0–1.5 | 0.8–1.4, 0.3–1.0
		T	3 | 7, 10	1.0 (0.9 ± 0.2) | 1.0 (1.0 ± 0.4), 1.3 (2.3 ± 3.5)	0.7–1.1 | 0.5–1.8, 0.4–12
		E	3 | 7, 10	0.2 (0.2 ± 0.1) | 0.2 (0.2 ± 0.1), 0.3 (0.3 ± 0.2)	0.2–0.3 | 0.1–0.4, 0.1–0.9
		X	3 | 7, 10	0.3 (0.4 ± 0.2) | 0.3 (0.4 ± 0.2), 0.4 (0.5 ± 0.4)	0.2–0.6 | 0.2–0.9, 0.1–1.5
Leech Lake (MN Cass)	2011	B	10	1.5 (1.4 ± 0.5)	0.5–2.2
		T	10	2.0 (4.5 ± 8.7)	0.6–29
		E	10	0.5 (0.5 ± 0.5)	0.0–1.7
		X	10	1.1 (1.2 ± 1.3)	0.4–4.6
Navajo Nation (NM McKinley)	2014 | 2015	B	2 | 13	0.9 (0.9 ± 0.04) | 1.3 (1.1 ± 0.3)	0.9–0.9 | 0.6–1.5
		T	2 | 13	0.6 (0.5 ± 0.04) | 0.9 (1.1 ± 0.7)	0.5–0.6 | 0.4–2.9
		E	2 | 13	0.1 (0.1 ± 0.0) | 0.2 (0.2 ± 0.1)	0.1–0.1 | 0.1–0.4
		X	2 | 13	0.3 (0.2 ± 0.03) | 0.4 (0.4 ± 0.2)	0.2–0.3 | 0.2–0.9
Navajo Nation (NM San Juan)	2010 | 2011	B	2 | 27	2.6 (2.1 ± 0.8) | 0.9 (1.0 ± 0.4)	1.5–2.6 | 0.4–2.1
		T	2 | 27	3.6 (3.3 ± 0.3) | 1.5 (1.8 ± 1.3)	3.1–3.6 | 0.4–6.4
		E	2 | 27	0.8 (0.8 ± 0.01) | 0.4 (0.4 ± 0.1)	0.8–0.8 | 0.2–0.7
		X	2 | 27	2.1 (2.0 ± 0.2) | 0.7 (0.7 ± 0.4)	1.9–2.1 | 0.2–1.4

^1^ If result is below sample screening level, not expected to cause adverse health effects from short-term exposures; * B—benzene CAS NO. 71-43-2; T—toluene CAS NO. 108-88-3; E—ethylbenzene CAS NO. 100-41-4; X—o-xylene CAS NO. 95-47-3; ^2^ sites 1 and 2 were 12 miles apart.

**Table 4:** Summary of outdoor air concentrations (ng/m^3^) of metals in PM_10_ by location and measurement year.

Metal (Sample Screening Level)	Tribe	Year	No. of Samples	Median (Mean ± Standard dev.)	Minimum–Maximum
Antimony (2000 ng/m^3^)	Morongo	2014	10	1.0 (1.1 ± 0.8)	0.1–2.7
	Nez Perce	2011	14	0.2 (0.5 ± 1.0)	0.1–4.0
	Red Lake (Site 1)	2010 | 2011	7 | 4	0.1 (0.3 ± 0.3) | 0.1 (0.1 ± 0.1)	0.1–0.8 | 0.0–0.1
	Red Lake (Site 2)	2011 | 2012	3 | 15	0.1 (0.1 ± 0.1) | 0.1 (0.1 ± 0.1)	0.0–0.1 | 0.0–0.4
	Leech Lake	2011 | 2012	9 | 1	0.2 (0.2 ± 0.2) | 0.2 (0.2 ± 0.0)	0.0–0.6 | 0.2–0.2
	Navajo Nation (NM McKinley)	2014 | 2015	2 | 13	0.2 (0.2 ± 0.0) | 0.3 (0.3 ± 0.2)	0.2–0.2 | 0.1–0.6
Arsenic (150 ng/m^3^)	Morongo	2014	10	0.4 (0.3 ± 0.2)	0.0–0.4
	Nez Perce	2011	14	0.2 (0.3 ± 0.2)	0.0–0.8
	Red Lake (Site 1)	2010 | 2011	7 | 4	0.2 (0.3 ± 0.2) | 0.1 (0.1 ± 0.1)	0.0–0.5 | 0.0–0.1
	Red Lake (Site 2)	2011 | 2012	3 | 15	0.2 (0.2 ± 0.2) | 0.2 (0.2 ± 0.1)	0.0–0.3 | 0.0–0.4
	Leech Lake	2011 | 2012	9 | 1	0.4 (0.4 ± 0.2) | 0.3 (0.3 ± 0.0)	0.1–0.7 | 0.3–0.3
	Navajo Nation (NM McKinley)	2014 | 2015	2 | 13	0.4 (0.4 ± 0.1) | 0.1 (0.2 ± 0.2)	0.3–0.4 | 0.0–0.9
Cadmium (30 ng/m^3^)	Morongo	2014	10	0.1 (0.1 ± 0.1)	0.0–0.1
	Nez Perce	2011	14	0.0 (0.02 ± 0.04)	0.0–0.1
	Red Lake (Site 1)	2010 | 2011	7 | 4	0.1 (0.1 ± 0.1) | 0.0 (0.0 ± 0.0)	0.0–0.2 | 0.0–0.0
	Leech Lake	2011 | 2012	9 | 1	0.1 (0.1 ± 0.04) | 0.1 (0.1 ± 0.0)	0.0–0.1 | 0.0–0.1
	Navajo Nation (NM McKinley)	2014 | 2015	2 | 13	0.1 (0.1 ± 0.1) | 0.0 (0.01 ± 0.03)	0.0–0.1 | 0.0–0.1
Chromium (580 ng/m^3^)	Morongo	2014	10	9.7 (9.2 ± 1.1)	7.6–11
	Nez Perce	2011	14	4.5 (4.6 ± 1.4)	2.9–8.5
	Red Lake (Site 1)	2010 | 2011	7 | 4	2.8 (2.9 ± 0.4) | 2.8 (2.6 ± 0.8)	2.5–3.8 | 1.7–3.7
	Red Lake (Site 2)	2011 | 2012	3 | 15	3.3 (3.8 ± 1.6) | 10 (7.8 ± 4.4)	2.5–5.6 | 2.3–13
	Leech Lake	2011 | 2012	9 | 1	11 (13 ± 5.4) | 18 (18 ± 0.0)	7.8–26 | 18–18
	Navajo Nation (NM McKinley)	2014 | 2015	2 | 13	3.1 (2.9 ± 0.35) | 3.6 (3.6 ± 0.60)	2.6–3.1 | 2.3–4.6
Cobalt (100 ng/m^3^)	Morongo	2014	10	0.2 (0.3 ± 0.1)	0.1–0.6
	Nez Perce	2011	14	0.5 (0.7 ± 0.5)	0.2–1.5
	Leech Lake	2011 | 2012	9 | 1	0.1 (0.1 ± 0.1) | 0.1 (0.1 ± 0.0)	0.0–0.2 | 0.1–0.1
	Navajo Nation (NM McKinley)	2014 | 2015	2 | 13	0.1 (0.1 ± 0.0) | 0.1 (0.1 ± 0.1)	0.1–0.1 | 0.0–0.1
Manganese (500 ng/m^3^)	Morongo	2014	10	12 (12 ± 7.0)	4.0–29
	Nez Perce	2011	14	24 (28 ± 19)	6.7–68
	Red Lake (Site 1)	2010 | 2011	7 | 4	2.3 (3.0 ± 3.0) | 1.1 (1.2 ± 1.3)	1.0–9.6 | 0.2–3.1
	Red Lake (Site 2)	2011 | 2012	3 | 15	2.2 (4.5 ± 5.8) | 7.0 (8.0 ± 5.7)	0.1–11 | 1.4–25
	Leech Lake	2011 | 2012	9 | 1	7.9 (8.8 ± 7.0) | 2.5 (2.5 ± 0.0)	3.5–26 | 2.5–2.5
	Navajo Nation (NM McKinley)	2014 | 2015	2 | 13	4.1 (4.0 ± 0.8) | 4.4 (4.5 ± 2.8)	3.0–4.1 | 0.3–9.9
Nickel (200 ng/m^3^)	Morongo	2014	10	0.8 (0.8 ± 0.4)	0.3–1.4
	Nez Perce	2011	14	0.8 (0.8 ± 0.4)	0.3–1.8
	Red Lake (Site 1)	2010 | 2011	7 | 4	0.4 (0.5 ± 0.4) | 0.3 (0.3 ± 0.13)	0.1–1.2 | 0.1–0.4
	Red Lake (Site 2)	2011 | 2012	3 | 15	0.2 (0.2 ± 0.2) | 0.4 (0.5 ± 0.2)	0.1–0.4 | 0.2–0.9
	Leech Lake	2011 | 2012	9 | 1	0.9 (1.6 ± 1.8) | 2.7 (2.7 ± 0.0)	0.3–6.2 | 2.7–2.7
	Navajo Nation (NM McKinley)	2014 |2015	2 | 13	0.20 (0.10 ± 0.14) | 0.40 (0.45 ± 0.22)	0–0.20 | 0–0.90
Selenium (20,000 ng/m^3^)	Morongo	2014	10	0.3 (0.3 ± 0.2)	0.0–0.7
	Red Lake (Site 1)	2010 | 2011	7 | 4	0.1 (0.21 ± 0.29) | 0.2 (0.1 ± 0.1)	0.0–0.8 | 0.0–0.2
	Red Lake (Site 2)	2011 | 2012	3 | 15	0.1 (0.3 ± 0.5) | 0.4 (0.3 ± 0.3)	0.0–0.9 | 0.0–0.8
	Leech Lake	2011 | 2012	9 | 1	0.2 (0.2 ± 0.2) | 0.0 (0.0 ± 0.0)	0.0–0.7 | 0.0–0.0
	Navajo Nation (NM McKinley)	2014 | 2015	2 | 13	0.2 (0.2 ± 0.1) | 0.1 (0.09 ± 0.1)	0.1–0.2 | 0.0–0.2

**Table 5:** Comparison of outdoor air concentrations by pollutant for schools on and off U.S. tribal lands.

Reference	Location—U.S. Tribe, State, County, and/or City	No. of Sampling Sites	Sampling Period	Summary Outdoor Air Concentration	Nearby Emission Source
Benzene					
U.S. EPA SATMI ^1^ 2016 [35]	CA, Los Angeles county, city of Lennox	1	20 samples: 8/2009–3/2010	Average 1.45 µg/m^3^ (range 0.521–3.58)	Multiple airports (closest ~2 miles) and surrounded by two interstate highways (closest <1 mile) and other roadway sources
U.S. EPA SATMI ^1^ 2016 [35]	CA, Los Angeles, city of Los Angeles	1	15 samples: 8/2009–3/2010	Average 1.82 µg/m^3^ (0.674–3.15)	Mix of small, industrial sources, and surrounded by two interstate highways (closest < 1 mile) and other roadway sources
Smith et al. 2006 [41]	TX, city of El Paso	22 schools	Two 7-day periods: 11–12/1999	Median west 2.28 µg/m^3^ (1.28–2.88) center 3.21 (1.66–4.99) east 1.83 (0.91–2.53)	Major highways due to border between USA and Mexico (e.g., one monitor within < 1 mile to international bridge)
U.S. EPA SATMI 2016 ^1^ [35]	Morongo (CA Riverside county)	1	11 samples, 2014	Average 1.60 µg/m^3^ (0.96–2.88)	3 miles from airport and concrete manufacturing
U.S. EPA SATMI 2016 ^1^ [35]	Rincon (CA San Diego county)	1	9 samples, 2012	Average 3.20 µg/m^3^ (1.60–8.94)	3 miles from airport
Metals in PM_10_—manganese					
U.S. EPA SATMI 2016 ^1^ [35]	MN, Hennepin county, city of Minneapolis	1	12 samples: 7/2009–10/2009	Average 8.68 ng/m^3^ (1.19–21.5)	Large municipal waste incinerator and coatings manufacturing facility
U.S. EPA SATMI 2016 ^1^ [35]	Red Lake (MN Beltrami county)	1	7 samples in 2010, 4 in site 1 and 3 in site 2 in 2011, 15 in 2012	Average 2010 3.0 ng/m^3^ (1.0–9.6) 2011	32 miles from airport
U.S. EPA SATMI 2016 ^1^ [35]	Leech Lake (MN Cass county)	1	9 samples in 2011, 1 in 2012	Average 2011 8.8 ng/m^3^ (3.5–26) 2012 2.5 (2.5–2.5)	4 miles from solid waste landfill
Metals in PM_10_—nickel					
U.S. EPA SATMI 2016 ^1^ [35]	MN, Hennepin county, city of Minneapolis	1	12 samples: 7/2009–10/2009	Average 0.79 ng/m^3^ (0.15–4.06)	Large municipal waste incinerator and coatings manufacturing facility
U.S. EPA SATMI 2016 ^1^ [35]	Red Lake (MN Beltrami county)	1	7 samples in 2010, 4 in site 1 and 3 in site 2 in 2011, 15 in 2012	Average 2010 0.5 (0.1–1.2) 2011 site 1 0.3 (0.1–0.4) 2011 site 2 0.2 (0.1–0.4) 2012 0.5 (0.2–0.9)	32 miles from airport
U.S. EPA SATMI 2016 ^1^ [35]	Leech Lake (MN Cass county)	1	9 samples in 2011, 1 in 2012	Average 2011 1.6 (0.3–6.2) 2012 2.7 (2.7–2.7)	4 miles from solid waste landfill
NO_2_					
Gonzales et al. 2005 [42]	TX, city of El Paso	20 schools at 4 monitoring stations	One 7-day period in winter (2/11–18) 1999	Average 20.6 ppb ± 7.1 (11.0–37.5)	Major highways due to border between USA and Mexico
Smith et al. 2006 [41]	TX, city of El Paso	22 schools (20 schools from Gonzales et al.’s pilot study + 2 additional schools) [42]	Two 7-day periods: 11–12/1999	Median west 20.6 ppb (11.8–27.3) center 28.3 ppb (18.5–37.0) east 18.5 ppb (10.6–27.5)	Major highways due to border between USA and Mexico
U.S. EPA SATMI 2016 ^1^ [35]	Morongo (CA Riverside county)	1	2015, 2016	2015 average 2.7 ± 3.0 ppb median 1.9 (0–42) 2016 average 3.3 ± 3.3 ppb median 2.4 (0–30)	3 miles from airport and concrete manufacturing

^1^ School Air Toxics Monitoring Initiative.

## Supplementary Materials

The following are available online at http://www.mdpi.com/1660-4601/16/1/36/s1, Table S1: Summary of outdoor air concentrations for NO_2_, O_3_, and PM_2.5_ around one school (Morongo in CA), Table S2: Summary of outdoor air concentrations (ng/m^3^) for other metals in PM_10_.

## References

[1] (2014). Indian Health Service, United States Department of Health and Human Services: Trends in Indian Health. 2014 Edition. https://www.ihs.gov/dps/index.cfm/publications/trends2014/.

[2] (2017). Centers for Disease Control and Prevention, United States Department of Health and Human Services: Summary health statistics: National Health Interview Survey: 2015. Table C-1. https://minorityhealth.hhs.gov/omh/browse.aspx?lvl=4&lvlid=30.

[3] Tulve N.S., Ruiz J.D.C., Lichtveld K., Darney S.P., Quackenboss J.J. (2016). Development of a conceptual framework depicting a child’s total (built, natural, social) environment in order to optimize health and well-being. J. Environ. Health Sci..

[4] Barros N., Tulve N., Heggem D., Bailey K. (2018). Review of built and natural environment stressors impacting American Indian/Alaska Native children. Rev. Environ. Health.

[5] Bruden D.J., Singleton R., Hawk C.S., Bulkow L.R., Bentley S., Anderson L.J., Herrmann L., Chikoyak L., Hennessy T.W. (2015). Eighteen years of respiratory syncytial virus surveillance: Changes in seasonality and hospitalization rates in southwestern Alaska Native children. Pediatr. Infect. Dis. J..

[6] Bulkow L.R., Singleton R.J., DeByle C., Miernyk K., Redding G., Hummel K.B., Chikoyak L., Hennessy T.W. (2012). Risk factors for hospitalization with lower respiratory tract infections in children in rural Alaska. Pediatrics.

[7] Gessner B.D. (2008). Lack of piped water and sewage services is associated with pediatric lower respiratory tract infection in Alaska. J. Pediatr..

[8] Hennessy T.W., Ritter T., Holman R.C., Bruden D.L., Yorita K.L., Bulkow L., Cheek J.E., Singleton R.J., Smith J. (2008). The relationship between in-home water service and the risk of respiratory tract, skin, and gastrointestinal tract infections among rural Alaska Natives. Am. J. Public Health.

[9] Morris K., Morganlander M., Coulehan J.L., Gahagen S., Arena V.C. (1990). Wood-burning stoves and lower respiratory tract infection in American Indian children. Am. J. Dis. Child..

[10] Reisman J., Rudolph K., Bruden D., Hurlburt D., Bruce M.G., Hennessy T. (2014). Risk factors for pneumococcal colonization of the nasopharynx in Alaska Native adults and children. J. Pediatr. Infect. Dis. Soc..

[11] Singleton R., Salkoski A.J., Bulkow L., Fish C., Dobson J., Albertson L., Skarada J., Kovesi T., McDonald C., Hennessy T.W. (2016). Housing characteristics and indoor air quality in households of Alaska Native children with chronic lung conditions. Indoor Air.

[12] Surdu S., Montoya L.D., Tarbell A., Carpenter D.O. (2006). Childhood asthma and indoor allergens in Native Americans in New York. Environ. Health.

[13] Robin L.F., Lees P.S.J., Winget M., Steinhoff M., Moulton L.H., Santosham M., Correa A. (1996). Wood-burning stoves and lower respiratory illnesses in Navajo children. Pediatr. Infect. Dis..

[14] Ware D.N., Lewis J., Hopkins S., Boyer B., Montrose L., Noonan C.W., Semmens E.O., Ward T.J. (2014). Household reporting of childhood respiratory health and air pollution in rural Alaska Native communities. Int. J. Circumpolar Health.

[15] Wenger J.D., Zulz T., Bruden D., Singleton R., Bruce M.G., Bulkow L., Parks D., Rudolph K., Hurlburt D., Ritter T. (2010). Invasive pneumococcal disease in Alaskan children: Impact of the seven-valent pneumococcal conjugate vaccine and the role of water supply. Pediatr. Infect. Dis. J..

[16] Petersen K.M., Singleton R.J., Leonard L. (2003). A qualitative study of the importance and etiology of chronic respiratory disease in Alaska native children. Alaska Med..

[17] Ernst P., Thomas D., Becklake M.R. (1986). Respiratory survey of North American Indian children living in proximity to an aluminum smelter. Am. Rev. Respir. Dis..

[18] Denham M., Schell L.M., Deane G., Gallo M.V., Ravenscroft J., DeCaprio A.P. (2005). Akwesasne Task Force on the Environment. Relationship of lead, mercury, mirex, dichlorodiphenyldichloroethylene, hexachlorobenzene, and polychlorinated biphenyls to timing of menarche among Akwesasne Mohawk girls. Pediatrics.

[19] Newman J., Aucompaugh A.G., Schell L.M., Denham M., DeCaprio A.P., Gallo M.V., Ravenscroft J., Kao C.C., Hanover M.R., David D. (2006). PCBs and cognitive functioning of Mohawk adolescents. Neurotoxicol. Teratol..

[20] Newman J., Gallo M.V., Schell L.M., DeCaprio A.P., Denham M., Deane G.D. (2009). Akwesasne Task Force on Environment. Analysis of PCB congeners related to cognitive functioning in adolescents. Neurotoxicology.

[21] Schell L.M., Gallo M.V., Denham M., Ravenscroft J., DeCaprio A.P., Carpenter D.O. (2008). Relationship of thyroid hormone levels to levels of polychlorinated biphenyls, lead, p,p’-DDE, and other toxicants in Akwesasne Mohawk youth. Environ. Health Perspect..

[22] Schell L.M., Gallo M.V., Ravenscroft J., DeCaprio A.P. (2009). Persistent organic pollutants and anti-thyroid peroxidase levels in Akwesasne Mohawk young adults. Environ. Res..

[23] Shields L.M., Wiese W., Skipper B., Charley B., Banally L. (1992). Navajo birth outcomes in the Shiprock uranium mining area. Health Phys..

[24] Orr M., Bove F., Kaye W., Stone M. (2002). Elevated birth defects in racial or ethnic minority children of women living near hazardous waste sites. Int. J. Hyg. Environ. Health.

[25] Gilbreath S., Kass P.H. (2006). Adverse birth outcomes associated with open dumpsites in Alaska Native villages. Am. J. Epidemiol..

[26] Gilbreath S., Kass P.H. (2006). Fetal and neonatal deaths and congenital anomalies associated with open dumpsites in Alaska Native villages. Int. J. Circumpolar Health.

[27] Newman J., Behforooz B., Khuzwayo A.G., Gallo M.V., Schell L.M. (2014). Akwesasne Task Force on the Environment. PCBs and ADHD in Mohawk adolescents. Neurotoxicol. Teratol..

[28] Schell L.M., Gallo M.V., DeCaprio A.P., Hubicki L., Denham M., Ravenscroft J. (2004). Thyroid function in relation to burden of PCBs, p,p’-DDE, HCB, mirex and lead among Akwesasne Mohawk youth: A preliminary study. Environ. Toxicol. Pharmacol..

[29] (2009). United States Environmental Protection Agency: School Air Toxics Ambient Monitoring Plan. https://www3.epa.gov/ttn/amtic/files/ambient/airtox/2009sat/SATMonitoringPlan.pdf.

[30] (2014). National Tribal Air Association: Status of Tribal Air Report. https://dl.dropboxusercontent.com/content_link/XlOchc2VOXP0E9cwPGsbSrZCGXsnoTW1mFg17Ms36oi2s0I8gbaC3ZlKMMtUnL95/file.

[31] (2017). United States Environmental Protection Agency: National Emissions Inventory (NEI). https://www.epa.gov/air-emissions-inventories/national-emissions-inventory-nei.

[32] (2017). United States Environmental Protection Agency: Criteria Air Pollutants. https://www.epa.gov/criteria-air-pollutants#self.

[33] (2017). United States Environmental Protection Agency: What are Hazardous Air Pollutants?. https://www.epa.gov/haps/what-are-hazardous-air-pollutants.

[34] (2009). United States Environmental Protection Agency: Quality Assurance Project Plan for the EPA School Air Toxics Monitoring Program. https://www3.epa.gov/ttn/amtic/files/ambient/airtox/2009sat/SATQAPP.pdf.

[35] (2016). United States Environmental Protection Agency: Assessing Outdoor Air Near Schools. https://www3.epa.gov/air/sat/.

[36] (2018). United States Environmental Protection Agency: Air Quality System (AQS). https://www.epa.gov/aqs.

[37] Omernik J.M. (1987). Ecoregions of the conterminous United States. Ann. Assoc. Am. Geogr..

[38] (1997). Commission for Environmental Cooperation: Ecological Regions of North America: Toward a Common Perspective. http://ecologicalregions.info/data/cec_na/CEC_NAeco.pdf.

[39] (2018). United States Environmental Protection Agency: Community-Focused Exposure and Risk Screening Tool (C-FERST). https://www.epa.gov/c-ferst.

[40] (2017). United States Environmental Protection Agency: Abandoned Mine Lands: Site Information. https://www.epa.gov/superfund/abandoned-mine-lands-site-information.

[41] Smith L., Mukerjee S., Gonzales M., Stallings C., Neas L., Norris G., Ozkaynak H. (2006). Use of GIS and ancillary variables to predict volatile organic compound and nitrogen dioxide levels at unmonitored locations. Atmos. Environ..

[42] Gonzales M., Qualls C., Hudgens E., Neas L. (2005). Characterization of a spatial gradient of nitrogen dioxide across a United States-Mexico border city during winter. Sci. Total Environ..

[43] (2017). United States Environmental Protection Agency: EPA’s Report on the Environment (ROE). https://cfpub.epa.gov/roe/indicator.cfm?i=71#2.

[44] (2014). Indian Health Service, United States Department of Health and Human Services: Regional Differences in Indian Health. 2012 Edition. https://www.ihs.gov/dps/includes/themes/responsive2017/display_objects/documents/RegionalDifferences2012Edition.pdf.

