# Supplementary material for: Outdoor Air Emissions, Land Use, and Land Cover around Schools on Tribal Lands
Source: Int J Environ Res Public Health. 2018 Dec 24;16(1):36. doi: 10.3390/ijerph16010036 (PMC6339187; doi:10.3390/ijerph16010036)
Supplement: Supplementary file 1 [file ijerph-16-00036-s001.pdf]

Supplementary Material

Table S1. Summary of outdoor air concentrations for NO_2, O_3, and $PM_{2.5}$ around one school (Morongo in CA).

Parameter	Year	Metric	No. of Samples	Mean ± Standard dev. (median)	Minimum–Maximum
NO_2 (ppb)	2015	Daily maximum 1-hour average ǀ observed values	8334 ǀ 8334	7.5 ± 5.1 (6.9) ǀ 2.7 ± 3.0 (1.9)	0.3–42 ǀ 0–42
	2016	Daily maximum 1-hour average ǀ observed values	8357 ǀ 8357	8.4 ± 5.0 (7.6) ǀ 3.3 ± 3.3 (2.4)	0.7–30 ǀ 0–30
O_3 (ppm)	2006	Daily maximum of observed hourly values (between 9:00AM and 8:00PM)	8019	0.06 ± 0.02 (0.05)	0.03–0.14
	2007		8744	0.07 ± 0.02 (0.06)	0.03–0.15
	2008		8053	0.07 ± 0.02 (0.06)	0.03–0.14
	2009		6548	0.07 ± 0.02 (0.07)	0.03–0.13
	2010		8719	0.06 ± 0.02 (0.06)	0.03–0.13
	2011		8685	0.06 ± 0.02 (0.06)	0.04–0.13
	2012		8774	0.07 ± 0.02 (0.06)	0.03–0.13
	2013		8757	0.07 ± 0.02 (0.06)	0.03–0.12
	2014		8642	0.07 ± 0.02 (0.06)	0.03–0.12
	2015		8136	0.06 ± 0.02 (0.06)	0.02–0.13
	2016		7712	0.06 ± 0.02 (0.06)	0.03–0.13
$PM_{2.5}$ (μg/m^3)	2006	Observed values	3124	9.8 ± 9.5 (7.0)	0–80
	2007		8590	14 ± 12 (11)	0–182
	2008		8468	12 ± 9.8 (9.0)	0–64
	2009		8327	11 ± 9.6 (9.0)	0–68
	2010		5023	11 ± 9.0 (9.0)	0–74
	2011		5277	14 ± 11 (13)	0–395
	2012		8230	9.7 ± 8.7 (7.0)	0–74
	2013		7044	8.0 ± 9.4 (6.0)	0–77
	2014		8428	11 ± 8.8 (9.0)	0–111
	2015		8615	9.8 ± 7.7 (8.0)	0–57
	2016		8363	11 ± 7.5 (9.0)	0–74

Table S2 Summary of outdoor air concentrations (ng/m^3) for other metals in PM_{10}.

Metal	Tribe	Year	No. of Samples	Median (Mean ± Standard dev.)	Minimum–Maximum
Beryllium	Morongo	2014	10	0.0 (0.01 ± 0.03)	0.0–0.1
	Nez Perce	2011	14	0.0 (0.03 ± 0.05)	0.0–0.1
	Red Lake (Site 1)	2010 ǀ 2011	7 ǀ 4	0.0 (0.0 ± 0.0) ǀ 0.0 (0.0 ± 0.0)	0.0–0.0 ǀ 0.0–0.0
	Red Lake (Site 2)	2011 ǀ 2012	3 ǀ 15	0.0 (0.0 ± 0.0) ǀ 0.0 (0.0 ± 0.0)	0.0–0.0 ǀ 0.0–0.0
	Leech Lake	2011 ǀ 2012	9 ǀ 1	0.0 (0.0 ± 0.01) ǀ 0.0 (0.0 ± 0.02)	0.0–0.0 ǀ 0.0–0.0
	Navajo Nation (NM McKinley)	2014 ǀ 2015	2 ǀ 13	0.0 (0.0 ± 0.0) ǀ 0.0 (0.0 ± 0.01)	0.0–0.0 ǀ 0.0–0.0
Lead	Morongo	2014	10	0.0 (0.0 ± 0.0)	0.0–0.0
	Nez Perce	2011	14	0.0 (0.0 ± 0.0)	0.0–0.2
	Red Lake (Site 1)	2010 ǀ 2011	7 ǀ 4	0.0 (0.0 ± 0.0) ǀ 0.0 (0.0 ± 0.0)	0.0–0.0 ǀ 0.0–0.0
	Red Lake (Site 2)	2011 ǀ 2012	3 ǀ 15	0.0 (0.0 ± 0.0) ǀ 0.0 (0.0 ± 0.0)	0.0–0.0 ǀ 0.0–0.0
	Leech Lake	2011 ǀ 2012	9 ǀ 1	0.0 (0.0 ± 0.0) ǀ 0.0 (0.0 ± 0.0)	0.0–0.0 ǀ 0.0–0.0
	Navajo Nation (NM McKinley)	2014 ǀ2015	2 ǀ 13	0.0 (0.0 ± 0.0) ǀ 0.0 (0.0 ± 0.0)	0.0–0.0 ǀ 0.0–0.0
Mercury	Morongo	2014	10	0.0 (0.0 ± 0.0)	0.0–0.0
	Nez Perce	2011	14	0.0 (0.0 ± 0.0)	0.0–0.0
	Red Lake (Site 1)	2010 ǀ 2011	7 ǀ 4	0.0 (0.0 ± 0.0) ǀ 0.0 (0.0 ± 0.0)	0.0–0.0 ǀ 0.0–0.0
	Red Lake (Site 2)	2011 ǀ 2012	3 ǀ 15	0.0 (0.0 ± 0.0) ǀ 0.0 (0.0 ± 0.0)	0.0–0.0 ǀ 0.0–0.0
	Leech Lake	2011 ǀ 2012	9 ǀ 1	0.0 (0.01 ± 0.03) ǀ 0.0 (0.0 ± 0.0)	0.0–0.1 ǀ 0.0–0.0
	Navajo Nation (NM McKinley)	2014 ǀ 2015	2 ǀ 13	0.0 (0.0 ± 0.0) ǀ 0.0 (0.0 ± 0.0)	0.0–0.0 ǀ 0.0–0.0
